# Fibroglandular tissue segmentation in breast MRI using vision transformers: a multi-institutional evaluation

**DOI:** 10.1038/s41598-023-41331-x

**Published:** 2023-08-30

**Authors:** Gustav Müller-Franzes, Fritz Müller-Franzes, Luisa Huck, Vanessa Raaff, Eva Kemmer, Firas Khader, Soroosh Tayebi Arasteh, Teresa Lemainque, Jakob Nikolas Kather, Sven Nebelung, Christiane Kuhl, Daniel Truhn

**Affiliations:** 1 Department of Diagnostic and Interventional Radiology, University Hospital RWTH, Aachen, Germany; 2 Else Kroener Fresenius Center for Digital Health, Technical University, Dresden, Germany; 3 Department of Medicine III, University Hospital RWTH, Aachen, Germany

**Keywords:** Medical research, Translational research

## Abstract

Accurate and automatic segmentation of fibroglandular tissue in breast MRI screening is essential for the quantification of breast density and background parenchymal enhancement. In this retrospective study, we developed and evaluated a transformer-based neural network for breast segmentation (TraBS) in multi-institutional MRI data, and compared its performance to the well established convolutional neural network nnUNet. TraBS and nnUNet were trained and tested on 200 internal and 40 external breast MRI examinations using manual segmentations generated by experienced human readers. Segmentation performance was assessed in terms of the Dice score and the average symmetric surface distance. The Dice score for nnUNet was lower than for TraBS on the internal testset (0.909 ± 0.069 versus 0.916 ± 0.067, P < 0.001) and on the external testset (0.824 ± 0.144 versus 0.864 ± 0.081, P = 0.004). Moreover, the average symmetric surface distance was higher (= worse) for nnUNet than for TraBS on the internal (0.657 ± 2.856 versus 0.548 ± 2.195, P = 0.001) and on the external testset (0.727 ± 0.620 versus 0.584 ± 0.413, P = 0.03). Our study demonstrates that transformer-based networks improve the quality of fibroglandular tissue segmentation in breast MRI compared to convolutional-based models like nnUNet. These findings might help to enhance the accuracy of breast density and parenchymal enhancement quantification in breast MRI screening.

## Introduction

Breast cancer is the most frequent type of cancer in the female population, and represents the second leading cause of death in the United States^1^ among women. New guidelines for breast cancer screening recommend the use of MRI for women with dense breast tissue^2,3^. Deep learning-based tools for the assessment of breast density on mammography have already been developed^4^, yet a consistent and reliable automated assessment of breast density—as the ratio of fibroglandular tissue (FGT) to the breast volume—on MRI examinations is still lacking. Besides breast density, background parenchymal enhancement (BPE)—the enhancement of fibroglandular tissue—has also emerged as a promising marker for the early detection of breast cancer^5,6^, however, reliable automated assessment of BPE is also lacking. The development of a machine learning algorithm capable of segmenting FGT is an important first step towards an automatic quantification of breast density and BPE in breast MRI examinations.

Several research studies have investigated this problem by training convolutional neural networks (CNNs) on manually segmented breast MRI examinations and evaluating their performance on single-center test sets^7–9^. The high level of agreement between human- and machine-generated segmentation maps in all of these publications demonstrates the potential of CNNs. However, there is an important impediment to the widespread introduction of such algorithms: MRI examinations are not standardized. Different clinical centers use diverse MRI protocols and sequences for the diagnosis of breast cancer. None of the studies we found tested their CNN architecture on independent data that did not belong to the institution where the algorithms were developed.

In addition, the development of robust models for breast MRI segmentation is challenging, especially in the presence of lesions, surgical scars, and breast implants^10,11^. The evaluation of segmentation models for breast MRI in the context of such potential confounders has received little attention. Previous studies have excluded breast implants^12,13^ or acknowledged the difficulty of accurately distinguishing between healthy and pathologic FGT^14^.

Transformer-based models have proven to be more robust, generalizable, and attack-proof than CNNs in other applications of medical image analysis^15,16^. They have achieved state-of-the-art results for natural language processing^17,18^, mainly because of their capability to handle long-term dependencies and self-supervised pre-training for downstream tasks.

Therefore, we aimed to develop and test a robust and accurate segmentation method based on the transformer architecture that could generalize well to multi-institutional data.

We compared our transformer-based breast segmentation (TraBS) model against the current state-of-the-art CNN-based model (nnUNet^19^) on both internal and external breast MRI datasets from Duke University^20^. Our hypotheses were that the new transformer-based model outperforms the current state of the art and that it generalizes better to external data.

## Results

This study included two datasets. An internal dataset (UKA) was used for training and testing TraBS and nnUNet. A second, external dataset (DUKE) was used to test the generalizability of both models. We tested the segmentation performance of TraBS and nnUNet in terms of the Dice score and the Average Symmetric Surface Distance (ASSD).

The Dice score quantifies the overlap between the automated segmentation and the ground truth, with values ranging from 0 to 1, where 1 represents a perfect overlap.

The Average Symmetric Surface Distance (ASSD) measures the average distance between the surfaces of the ground truth and the automated segmentation, where 0 mm represents a perfect alignment with the ground truth.

### Patient characteristics

The UKA dataset included 200 female patients, with a mean age of 56 ± 10 years (range 19–91) and a mean weight of 75 ± 27 kg. The DUKE dataset included 40 female patients with a mean age of 53 ± 11 years (range 22–90) with a mean weight of 76 ± 18 kg. In UKA and DUKE datasets, 9 women had breast implants, while in the DUKE dataset, no woman had a breast implant. Mean FGT volume and density were 117 ± 91 mm^3^ and 18 ± 14% for the UKA dataset and 114 ± 61 mm^3^ and 12 ± 7% for the DUKE dataset, respectively.

### Transformer outperforms CNNs on the internal dataset

#### Overall segmentation performance

nnUNet achieved a mean Dice score of 0.909 ± 0.069 for the FGT segmentation on our internal dataset, see Table 1. Our TraBS model achieved a higher mean Dice score of 0.916 ± 0.067, P < 0.001.

**Table 1:** Comparison of the dice score (DSC, higher is better), average symmetric surface distance (ASSD, lower is better), Pearson correlation coefficients between quantitative estimation by radiologists and neural network of the Breast Density (ρ_Dense_, higher is better) and background parenchymal enhancement (ρ_BPE_, higher is better) on the UKA dataset.

	DSC	ASSD (mm)	ρ_Dense_	ρ_BPE_
nnUNet	0.909 ± 0.069	0.657 ± 2.856	0.995	0.950
TraBS (ours)	**0.916 ± 0.067**	**0.548 ± 2.195**	**0.996**	**0.992**

The best performance is denoted in bold.

TraBS also demonstrated a lower ASSD (0.548 ± 2.195) than nnUNet (0.657 ± 2.856, P = 0.001), indicating, that finer details are more accurately assessed by TraBS.

**Figure 1:**
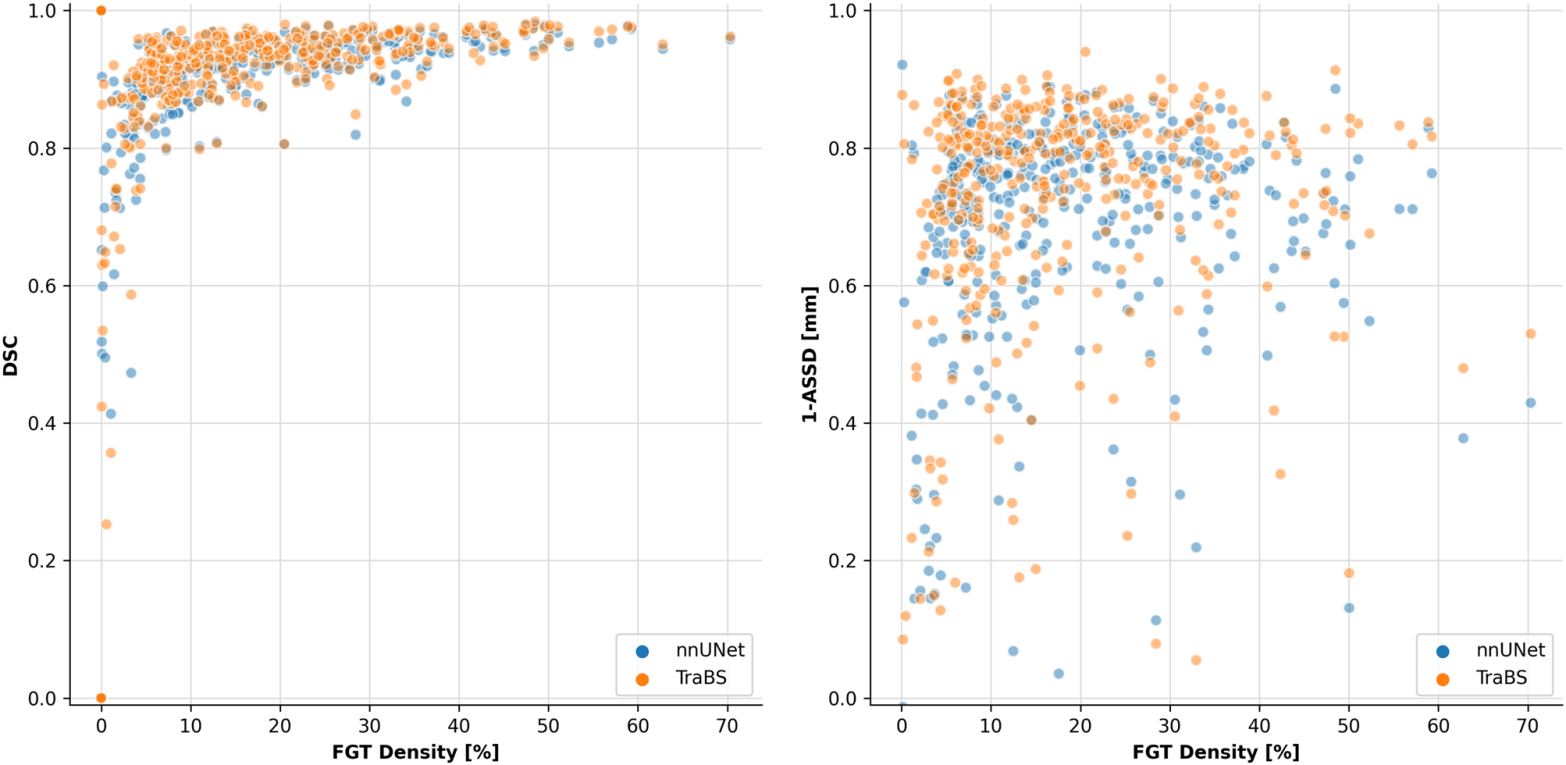
Dice similarity coefficient (DSC) and average symmetric surface distance (ASSD) between the automated and manual segmentations for all examined neural network architectures. Independent of the neural network used, DSC was lower in examinations of low-density breasts, while ASSD was not influenced by breast density.

By trend, segmentation performance as measured by Dice score and ASSD was lower for both models when breasts were less dense, i.e., when the fractional volume of FGT within the breast was lower, see Fig. 1.

#### Fine details and overall structure are better captured by TraBS

**Figure 2:**
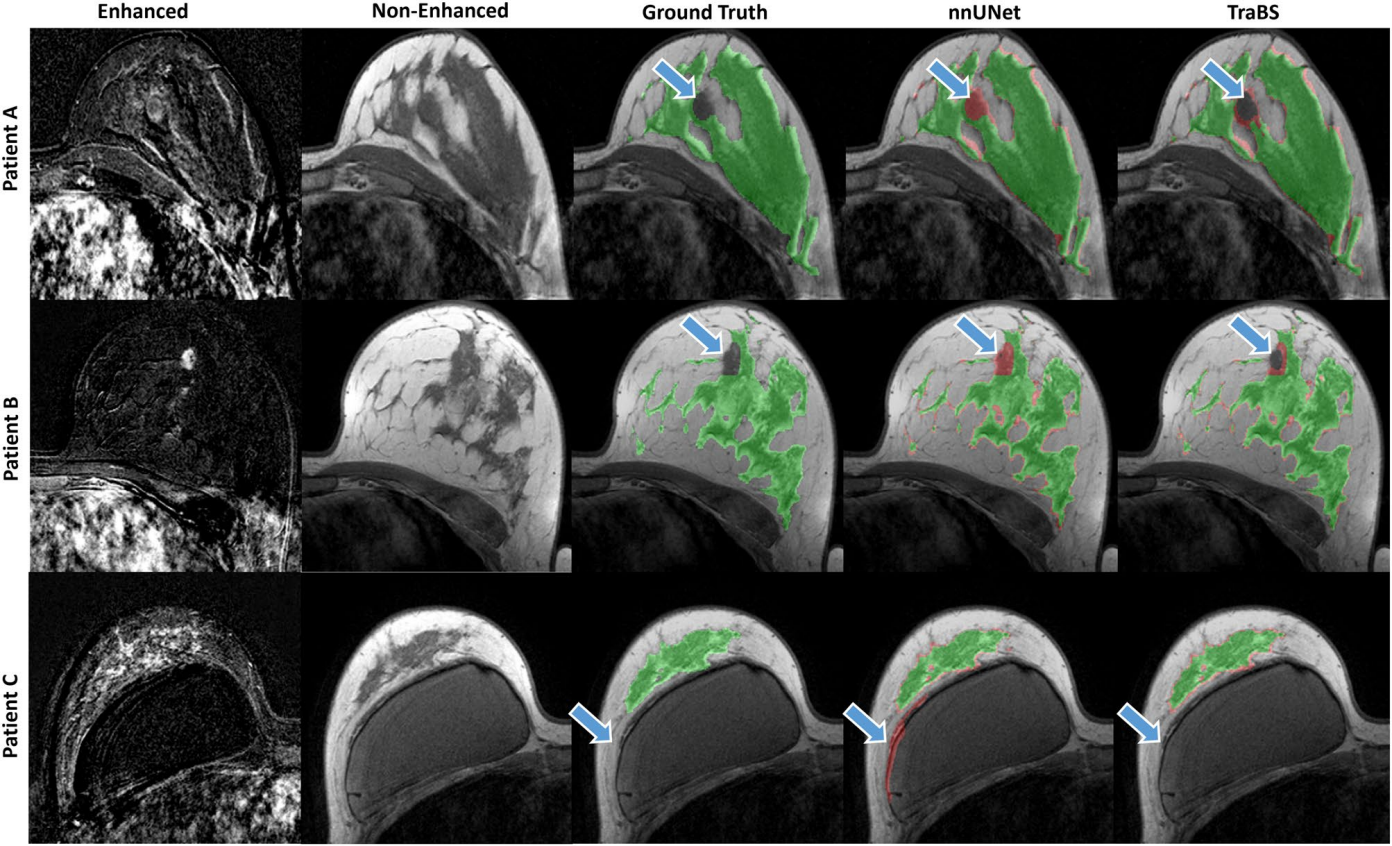
Sample MRI examinations of the internal UKA dataset. The two leftmost columns show the contrast-enhanced subtraction and non-enhanced T1-weighted image. The third column shows the ground truth segmentation by the radiologists and the two remaining columns show the segmentations by the neural networks. Correct segmentations are displayed in green and incorrectly labeled regions in red. Blue arrows denote challenging regions such as lesions (Patient A and B) or breast implants (Patient C).

In addition to quantitative assessment, an expert radiologist visually assessed the segmentation quality and found that TraBS performed better in capturing both overall structure and fine details compared to nnUNet. Specifically, TraBS was better at differentiating between breast implants and FGT and distinguishing between lesions and normal breast tissue, as shown in Fig. 2.

#### Transformer-based segmentation translates to more accurate clinical measures

To investigate how the segmentation performance relates to clinical measurements used to assess patients’ risks such as breast density, we examined the correlation between such measures when calculated based on the ground truth segmentation and on the automated segmentation. Both nnUNet and TraBS demonstrated an almost perfect correlation to the manually derived breast density and BPE, as shown in Table 1. Although the correlations were almost perfect for both models, TraBS showed a higher density correlation (ρ = 0.996 with 95% confidence interval [0.995, 0.997] vs ρ = 0.995 [0.994, 0.996]; P = 0.11) and BPE correlation (ρ = 0.992 [0.990, 0.994] vs ρ = 0.950 [0.939, 0.959]; P = 0.06).

### Transformer outperforms CNN on the external datasets

#### Overall segmentation performance

Applying the models to unseen external datasets with differing MRI sequence protocols resulted in overall lower performance (Table 2).

**Table 2:** Comparison of the dice score (DSC, higher is better), average symmetric surface distance (ASSD, lower is better), Pearson correlation coefficients between quantitative estimation by radiologists and neural network of the Breast Density (ρ_Dense_, higher is better) and background parenchymal enhancement (ρ_BPE_, higher is better) in the external DUKE dataset.

	DSC	ASSD (mm)	ρ_Dense_	ρ_BPE_
nnUNet	0.824 ± 0.144	0.727 ± 0.620	0.901	0.979
TraBS (ours)	**0.864 ± 0.081**	**0.584 ± 0.413**	**0.955**	**0.987**

The best performance is denoted in bold.

However, TraBS still performed better than nnUNet, achieving a mean Dice score of 0.864 ± 0.081 for the DUKE dataset, compared to 0.824 ± 0.144 (P = 0.004) for nnUNet. Similarly, ASSD was higher (= worse) for nnUNet (0.727 ± 0.620) than for TraBS (0.584 ± 0.413, P = 0.034).

#### Fine details and overall structure are better captured by TraBS

**Figure 3:**
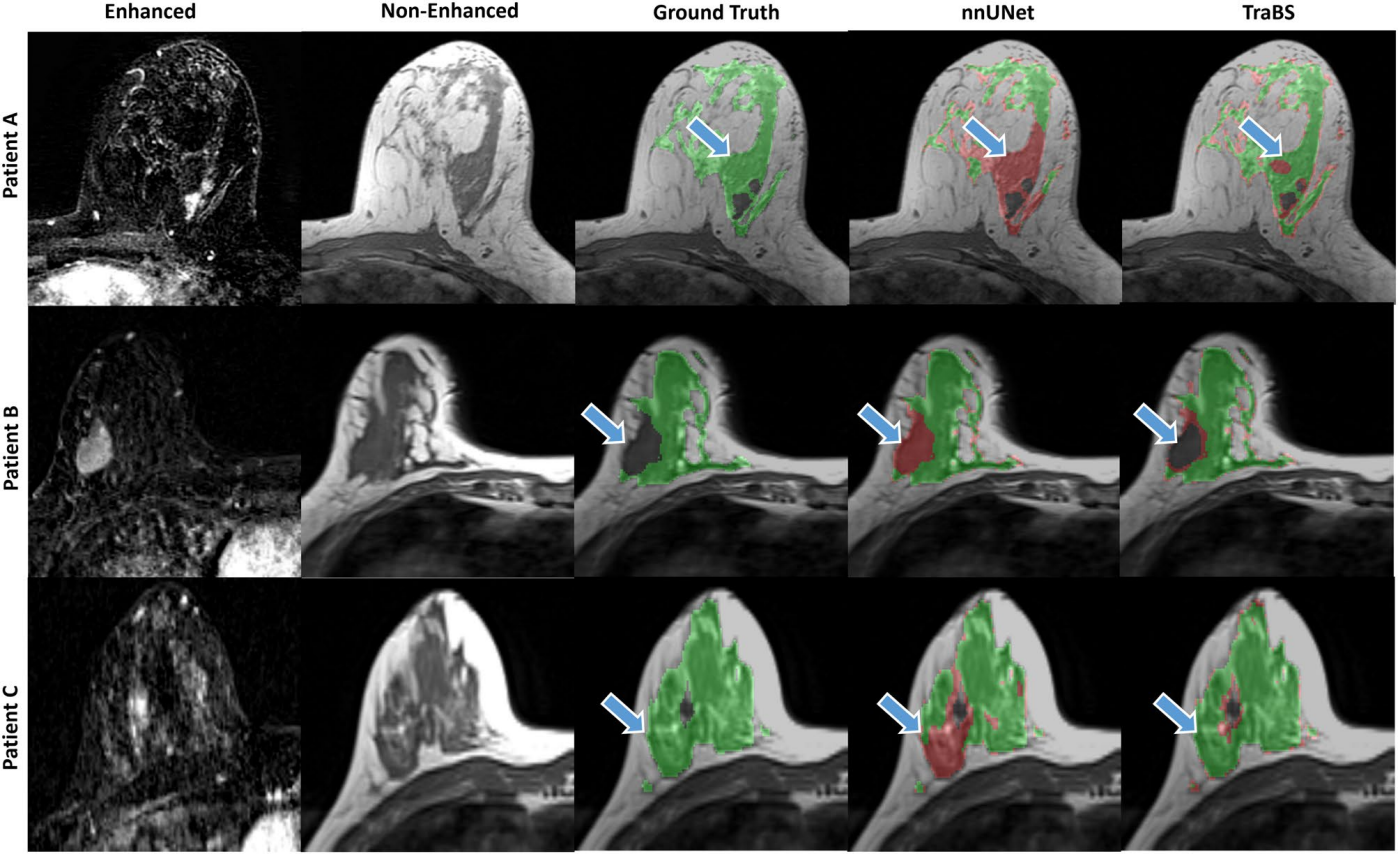
Sample MRI examinations of the external DUKE dataset. The two leftmost columns show the contrast-enhanced subtraction and non-enhanced T1-weighted image. The third column shows the ground truth segmentation by the radiologists and the two remaining columns show the segmentations by the neural networks. Correct segmentations are displayed in green and incorrectly labeled regions in red. Blue arrows denote challenging regions such as lesions (Patient A and B) or breast implants (Patient C).

Visual inspection of the segmentations in the external datasets confirmed the superior performance of TraBS, as it was better able to capture fine details and overall structure compared to nnUNet. Sample images are given in Fig. 3.

### Transformer-based segmentation translates to more accurate clinical measures

Despite the limited overall segmentation quality on the external DUKE dataset, both TraBS and nnUNet still demonstrated good correlations with manual segmentations for breast density and BPE (Table 2). However, TraBS achieved a higher density correlation (ρ = 0.955 [0.931, 0.971] vs ρ = 0.901 [0.849, 0.935]; P = 0.007) and BPE correlation (ρ = 0.987 [0.980, 0.992] vs ρ = 0.979 [0.967, 0.987]; P = 0.24) than nnUNet.

## Discussion

In this study, we propose a novel network architecture, TraBS, for segmenting fibroglandular tissue (FGT) in breast MRI images. We demonstrate that TraBS outperforms the previous state of the art in both internal and external validation sets. Breast density and BPE are important factors in determining patients’ cancer risk. Therefore, accurate and reliable methods for the automated extraction of quantitative markers such as breast density and BPE are needed. Our research contributes to the field in four aspects.

First, all groups who have applied neural networks on FGT segmentation have only evaluated their algorithms on internal test sets, i.e., examinations that are similar in appearance to the examinations upon which the algorithm was trained, see Table 3 for an overview of previous research. This is a shortcoming that needs to be addressed in view of the plethora of MRI scanner protocols that are currently in clinical use. We addressed this gap by evaluating our proposed TraBS model on an external dataset and we demonstrated that the new transformer-based architecture exhibits better generalization performance compared to nnUNet.

**Table 3:** Comparison of studies using neural networks for fibroglandular tissue (FGT) segmentation in MRI. The number of patients refers to the test set(s).

Study	Patients	Region	DSC FGT	ρ_FGT_	ρ_Dense_	ρ_BPE_
Ivanovska^31^	40	Germany	0.93 ± 0.01	–	–	–
Huo^7^	100	China	0.88 ± 0.08	0.972	0.981	–
Ma^32^	100	-	0.87 ± 0.07	–	–	0.63*
Dalmis^8^	66	Netherlands	0.85 ± 0.09	–	0.974	–
Nam ^12^	200	Korea	0.85 ± 0.11	0.93**	–	–
Zhang^9^	28	Asia	0.83 ± 0.06	0.97–0.99	–	–
Zhang^33^	40	–	0.81 ± 0.08	0.956	–	–
Ha^34^	137	USA	0.81	0.998	–	0.955
Ours	200	Germany	0.92 ± 0.07	0.996	0.996	0.992
	40	USA	0.86 ± 0.08	0.945	0.955	0.987

The agreement between the manual and neural network segmentation was measured based on the Dice Similarity Coefficient (DSC), the Pearson correlation coefficient (ρ_FGT_) of the FGT volume, the breast density (ρ_Dense_), and the correlation (ρ_BPE_) of the background parenchymal enhancement (BPE). *BPE was estimated qualitatively by radiologists, **Spearman correlation coefficient.

Second, we examined the Dice score as a function of breast density and found that lower FGT density results in a lower Dice score. This partly explains the spread of reported Dice scores in the literature (Table 3), as the test set that is used for evaluation has a large effect on the Dice metric: if segmentation algorithms are tested on breast MRI examinations with high amounts of FGT, Dice scores tend to be higher. This is an important finding for future studies and therefore, we suggest that future works on FGT segmentation should contain a report about the mean FGT density of the test set or a graph similar to Fig. 1.

Third, we make the manual segmentations for the DUKE data publicly available to serve as a reference standard for future evaluations. This can potentially contribute to independent external evaluations of segmentation algorithms for breast MRI.

Finally, we demonstrate the overall better performance of our transformer-based model TraBS as compared to the previous state-of-the-art architecture for breast tissue segmentation in all selected performance metrics. We make our code publicly available, alongside the trained model, to further advance the field and to bridge the gap to clinical application.

Our work has limitations that relate to the fact that manual segmentations are extremely time-consuming to obtain: First, even though we evaluated the model on external test data, we did not include any external training data. Consequently, the segmentation performance decreases when applied to external data and even though TraBS is more robust to domain shift, its performance could be increased by including additional multi-domain data during training. Future studies should focus on this to make the segmentation performance more robust so that the model can be applied at multiple centers. The task is particularly challenging because breast MRI protocols differ substantially between institutions including the choice of sequences, fat suppression and scan orientation. Second, we included only 40 external examinations as test cases from a single external institution. Even though this represents progress compared to previous research, the database for a broad multi-institutional study can and should be extended to provide a global perspective on the performance of underrepresented patient groups. Third, our data represented only a small sample of all possible MRI scanners and protocols. This may limit the direct applicability of automatic FGT segmentation and needs to be investigated for specific MRI configurations in further studies. Fourth, we did not investigate inter-rater variability due to the lack of multiple segmentations by multiple readers on the same examinations. This should be done by future studies to evaluate the accuracy of the human-generated segmentations which served as ground truth.

## Conclusion

In conclusion, our proposed TraBS network demonstrates excellent performance in segmenting FGT in breast MRI images. This paves the way for routine automated FGT segmentation and automatic quantification of breast density and BPE.

## Materials and methods

### Ethics statement

The Medical Ethics Committee of the Medical Faculty of RWTH Aachen University approved the study (EK028/19) and waived the need for informed consent. All methods were carried out in accordance with the Declaration of Helsinki.

### Datasets

In this retrospective study, two breast MRI datasets were used which we will refer to as “UKA” and “DUKE”. First, UKA was collected between 2010 and 2019 at the University Hospital Aachen, Germany^21^. UKA comprises a total of 9751 breast MRI examinations of 5086 women. We separated the examinations into two subsets, which were likely to include either malignant or benign examinations based on the BI-RADS scores. Among both sets, a total of 200 examinations from 200 women were randomly chosen, comprising 104 carcinomas, 55 fibroadenomas, and 41 lesion-free examinations. Dynamic contrast-enhanced (DCE)-MRI of the breasts was conducted according to a standardized protocol^22^ at a 1.5-T (Achieva and Ingenia; Philips Medical Systems) with a double-breast four-element surface coil (Invivo). Two paddles were used to immobilize the breast in the craniocaudal direction (Noras). Please refer to Table 4 for a detailed description of the acquisition parameters.

**Table 4:** Image acquisition parameters.

	UKA	DUKE		
Country	Germany	USA		
Orientation	Axial	Axial		
Scanner	Philips	GE, Siemens		
Field strength (T)	1.5	1.5, 3		
Acquisition	2010–2019	2000–2014		

	DCE	T2	DCE	T1
Acquisition type	2D	2D	3D	2D, 3D
Echo type	GR	SE	GR	SE, GR
Fat suppression	No	No	Yes	No
TR (ms)	264 ± 22	4008 ± 201	GR:5.0 ± 0.7	2D-SE:605 ± 66 2D-RM:5090 ± 191 3D-GR:6.7 ± 0.7
TE (ms)	4.6 ± 0.1	110	GR:2.1 ± 0.5	2D-SE:9.9 ± 1.2 3D-GR:3.5 ± 0.8
Flip angle (°)	90 ± 3	90	10 ± 0.5	97 ± 44
Slice thickness (mm)	3.1 ± 0.2	3.1 ± 0.2	1.2 ± 0.3	2.3 ± 0.7
Number of slices	28 ± 2	33 ± 2	170 ± 23	80 ± 51
Matrix X	520 ± 22	535 ± 39	487 ± 43	438 ± 103
Matrix Y	520 ± 22	535 ± 39	487 ± 43	438 ± 103
Field of view X (mm)	333 ± 26	332 ± 26	347 ± 25	345 ± 25
Field of view Y (mm)	333 ± 26	332 ± 26	347 ± 25	345 ± 25

*NA* not available, *GR* gradient echo, *SE* spin echo, *DCE* dynamic contrast enhancement.

Second, DUKE was collected between 2000 and 2014 at the Duke Hospital, USA, and is publicly available^20^. All 922 cases have biopsy-confirmed invasive breast cancer and were acquired using a 1.5 Tesla or 3.0 Tesla scanner from General Electric or Siemens. The MRI protocol consisted of a T1-weighted fat-suppressed sequence (one pre-contrast, and four post-contrast scans) and a non-fat-suppressed T1-weighted sequence. For evaluation, 40 examinations were randomly selected and manually segmented as described below.

### Ground truth segmentation

Both the whole breast volume and the fibroglandular tissue were segmented by F.M. and E.K. using the software ITK-SNAP^23^. The segmentations were reviewed by L.H. and V.R., two radiologists with six and three years of experience in breast MRI. Any discrepancies between the raters were discussed and resolved in consensus. Segmentation masks were generated for the UKA subset of 200 MRI examinations and 40 randomly sampled cases of DUKE, respectively. The breast outline was defined as the tissue volume located anterior to the pectoralis muscle. Sample manual segmentations are shown in Supplemental Figs. S1 and S2.

### Automatic segmentation pipeline

**Figure 4:**
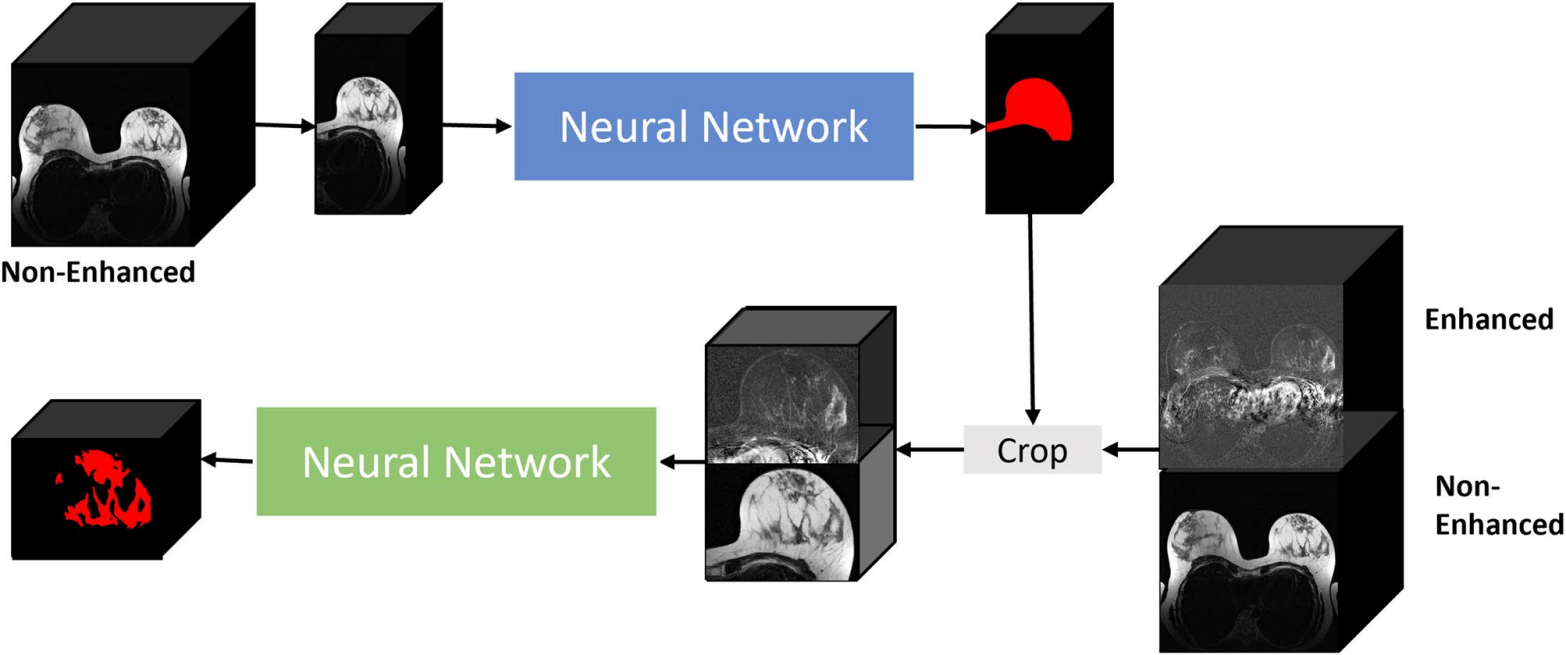
Illustration of the segmentation framework. The framework was trained on UKA data. The first neural network used non-enhanced, non-fat-suppressed T1- and T2-weighted sequences to segment the whole breast. This segmentation was subsequently used to crop the subtraction image of the contrast-enhanced, T1-weighted sequence as well as the non-enhanced sequences. Based on the cropped images, the second neural network created a segmentation mask of the fibroglandular tissue. For inference of whole breast segmentation masks from the DUKE dataset, only non-fat-saturated T1-weighted sequences were used as inputs for the first neural network, as T2-weighted sequences were only available for the UKA dataset. Please note that the networks processed all inputs as 3D volumes.

The segmentation pipeline comprised two consecutive stages. In the first stage, the entire breast was segmented, while in the second stage only the FGT was segmented (Fig. 4). In both stages, the use of a neural network was possible, however, the manual (ground truth) segmentations were used in the first stage with the rationale that we want to compare the network architectures for FGT segmentation only.

For the second stage of the segmentation pipeline, the segmentation masks from the first stage were used to create cropped images of the left and right breast. The non-enhanced and the contrast-enhanced images were stacked along the channel dimension and both breast sides were subsequently fed into the neural network. The intensity distributions of all images were z-score normalized (mean = 0, standard deviation = 1). The segmentation pipeline was implemented with PyTorch^24^ on a computer equipped with an NVIDIA GeForce RTX 3090.

### Model architecture

**Figure 5:**
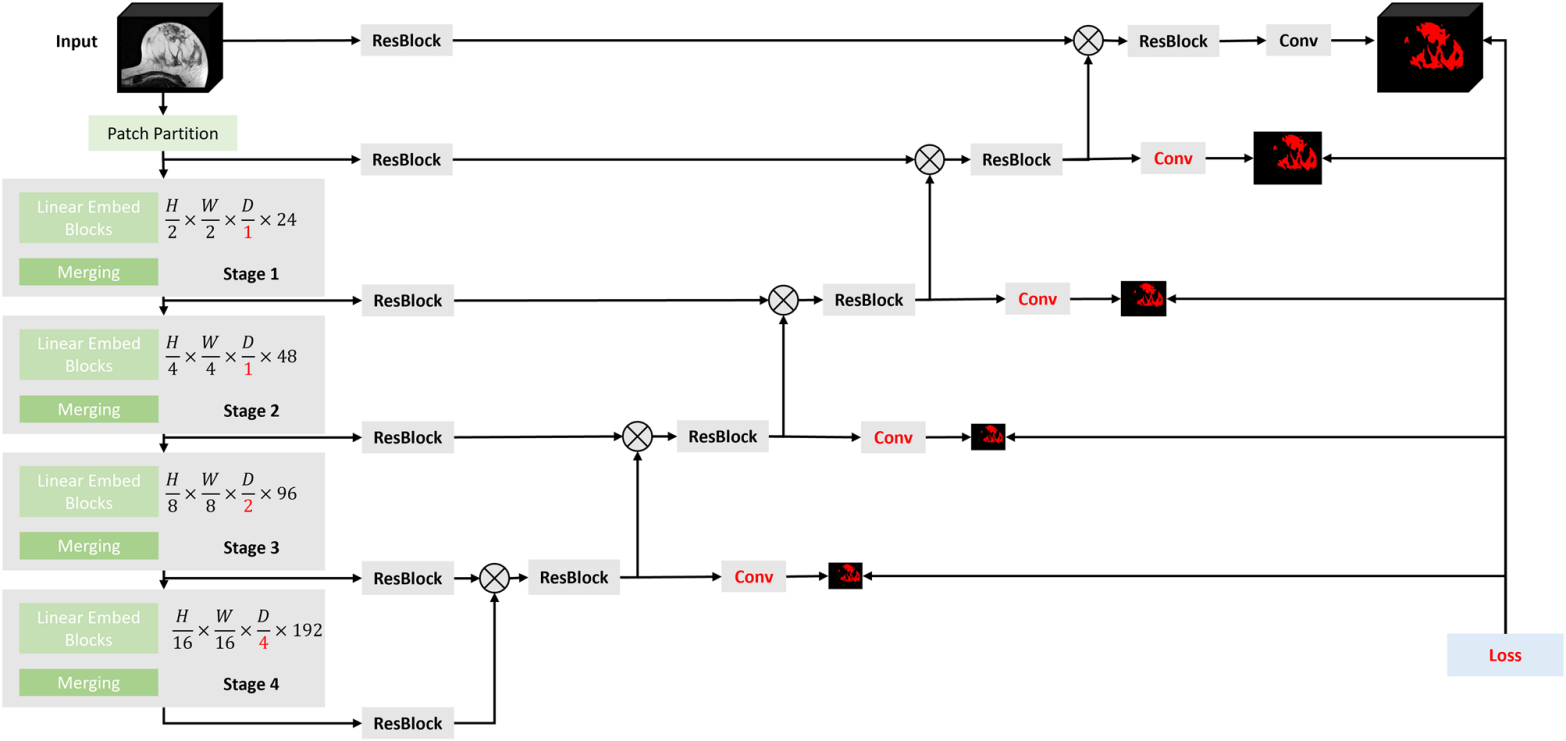
Illustration of the proposed TraBS model architecture. Non-isotropic kernels and strides were used in the first two stages in order for the depth to remain constant. Deep supervision was added for the lower-resolution layers. Changes from the original SwinUNETR have been marked in red. Please refer to the SwinUNETR publication^25^ for an in-depth explanation.

In the following, we refer to our new transformer-based model as TraBS (SwinTransformer for fibroglandular Breast tissue Segmentation). TraBS was built upon SwinUNETR^25^ with 2, 4, and 8 heads and 24, 48, 96, and 192 embedding features in stages 1 to 4. Inspired by the nnUNet to handle typically non-isotopic resolutions in MRI images, we replaced the uniform 2 × 2 × 2 patch sizes and 3 × 3 × 3 kernels in the two up-most layers with non-isotropic 1 × 2 × 2 patches and 1 × 3 × 3 kernels. In addition, 1 × 1 × 1 convolutions were added to supervise the deeper layers (Fig. 5).

We employed the state-of-the-art nnUNet^19^ as a baseline. The model had two max-pooling layers with 1 × 2 × 2 strides and 1 × 3 × 3 kernels, followed by two max-pooling layers with 2 × 2 × 2 strides and 3 × 3 × 3 kernels. This was motivated by a previous publication for FGT segmentation^7^.

### Training and testing of the framework

The UKA subset was randomly divided into training and test sets using five-fold cross-validation. The training set within each fold was further subdivided into a dedicated training set (80%) and a validation set (20%). The training of the FGT segmentation models was performed for each of the five folds with the manual segmentation masks as ground truth. AdamW with a learning rate of 0.0001 was used to optimize the sum of DiceLoss and CrossEntropy, following previous recommendations for medical image segmentation^26^. The loss function was additionally calculated at the lower resolutions of the decoder path (Multi-Scale Supervision) in the TraBS model, following the nnUNet implementations. Using early stopping, training of each model was halted as soon as the loss within the validation set did not decrease within 30 epochs.

### Data augmentation

To increase the diversity of the training set and thus prevent overfitting, the following data augmentation operations from the TorchIO framework^27^ were applied: flipping, affine transformation, ghosting, Gaussian noise, blurring, bias field, and gamma augmentation. During training, a random region of 256 × 256 × 32 voxels within the left and right breast was selected. A sliding window of 256 × 256 × 32 voxels with an overlap of 50% was used during inference. Random-flip along all axes was used as test-time augmentation. The source code is publicly available at https://github.com/mueller-franzes/TraBS.

### Statistical analysis

We performed a five-fold cross-validation on the internal UKA dataset to examine the performance of the models on unseen test data. For the external DUKE dataset, an ensemble of the five FGT segmentation models from the cross-validation training was applied. Majority voting was used to combine the five segmentation masks. Segmentation performance was assessed by calculating the Dice similarity coefficient (DSC)^28^ and Average Symmetric Surface Distance (ASSD)^29^. Breast density and the BPE are both clinically relevant metrics related to breast cancer risk^5,6^. Their quantitative assessment depends on the FGT segmentation. Therefore, we measured these two metrics both for the manual and the automated segmentations and calculated the Pearson correlation coefficients between manually and automatically derived metrics. Note, that the BPE was defined as the percentage change of the FGT between the post- and pre-contrast image. Bootstrapping was employed to calculate confidence intervals and permutation testing was used to calculate p-values. Following the guidance of Amrhein et al.^30^, we did not set thresholds for statistical significance when interpreting the p-values.

### Supplementary Information

Supplementary Information.

## Data Availability

The DUKE dataset analysed during the current study is available in The Cancer Imaging Archive, https://doi.org/10.7937/TCIA.e3sv-re93. The UKA dataset analysed during the current study is available from the corresponding author on reasonable request.
