# Supplementary material for: Fibroglandular tissue segmentation in breast MRI using vision transformers: a multi-institutional evaluation
Source: Sci Rep. 2023 Aug 30;13:14207. doi: 10.1038/s41598-023-41331-x (PMC10468506; doi:10.1038/s41598-023-41331-x)
Supplement: Supplementary file 1 — Supplementary Information. [file 41598_2023_41331_MOESM1_ESM.docx]

# Supplemental Material

**Table S1**: Mean DSC values within the five-fold cross-validation.

|  | Fold 1 | Fold 2 | Fold 3 | Fold 4 | Fold 5 |
| --- | --- | --- | --- | --- | --- |
| nnUNet | 0.887±0.100 | 0.919±0.039 | 0.925±0.032 | 0.904±0.065 | 0.913±0.075 |
| TraBS (ours) | **0.889±0.104** | **0.923±0.040** | **0.934±0.030** | **0.913±0.052** | **0.922±0.068** |


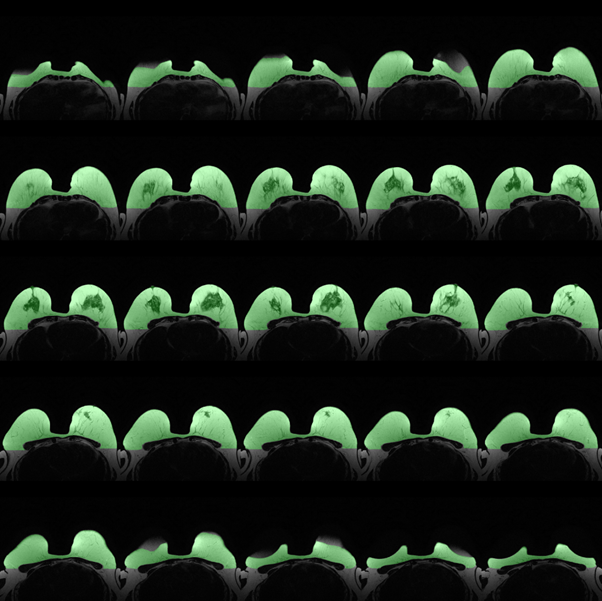


**Supplemental Figure S1**: Illustration of the manual breast volume segmentation in the UKA dataset.


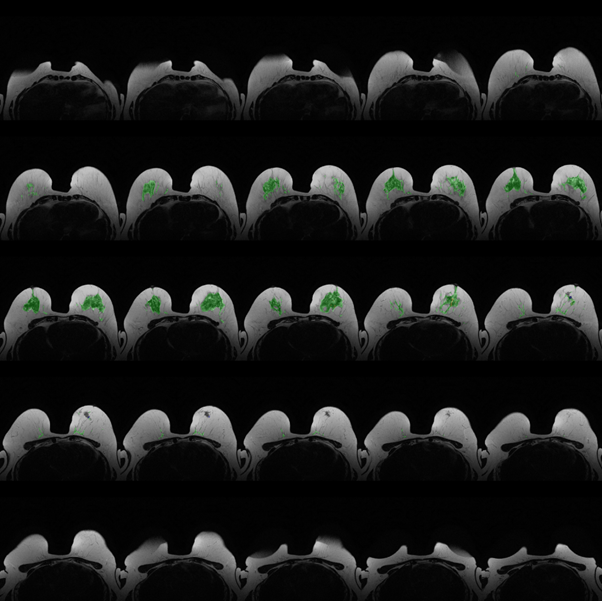


**Supplemental Figure S2**: Illustration of the manual fibroglandular tissue segmentation in the UKA dataset.
